# Safety of Single-Dose Primaquine in G6PD-Deficient and G6PD-Normal Males in Mali Without Malaria: An Open-Label, Phase 1, Dose-Adjustment Trial

**DOI:** 10.1093/infdis/jiy014

**Published:** 2018-01-12

**Authors:** Ingrid Chen, Halimatou Diawara, Almahamoudou Mahamar, Koualy Sanogo, Sekouba Keita, Daouda Kone, Kalifa Diarra, Moussa Djimde, Mohamed Keita, Joelle Brown, Michelle E Roh, Jimee Hwang, Helmi Pett, Maxwell Murphy, Mikko Niemi, Bryan Greenhouse, Teun Bousema, Roly Gosling, Alassane Dicko

**Affiliations:** 1 Malaria Elimination Initiative, Global Health Group, San Francisco; 2 Department of Epidemiology and Biostatistics, San Francisco; 3 School of Medicine, University of California, San Francisco; 4 President’s Malaria Initiative, Malaria Branch, Division of Parasitic Diseases and Malaria, Centers for Disease Control and Prevention, Atlanta, Georgia; 5 Malaria Research and Training Centre, Faculty of Pharmacy and Faculty of Medicine and Dentistry, University of Science, Techniques and Technologies of Bamako; 6 National University Hospital of Point G, Bamako, Mali; 7 Department of Medical Microbiology, Radboud University Medical Center, Nijmegen, the Netherlands; 8 Department of Clinical Pharmacology, University of Helsinki and Helsinki University Hospital, Finland; 9 Department of Immunology and Infection, London School of Hygiene and Tropical Medicine, United Kingdom

**Keywords:** Primaquine, Plasmodium falciparum, malaria, transmission, G6PD deficiency, drug safety, hemolysis, mass drug administration

## Abstract

**Background:**

The World Health Organization recommendation on the use of a single low dose of primaquine (SLD-PQ) to reduce *Plasmodium falciparum* malaria transmission requires more safety data.

**Methods:**

We conducted an open-label, nonrandomized, dose-adjustment trial of the safety of 3 single doses of primaquine in glucose-6-phosphate dehydrogenase (G6PD)-deficient adult males in Mali, followed by an assessment of safety in G6PD-deficient boys aged 11–17 years and those aged 5–10 years, including G6PD-normal control groups. The primary outcome was the greatest within-person percentage drop in hemoglobin concentration within 10 days after treatment.

**Results:**

Fifty-one participants were included in analysis. G6PD-deficient adult males received 0.40, 0.45, or 0.50 mg/kg of SLD-PQ. G6PD-deficient boys received 0.40 mg/kg of SLD-PQ. There was no evidence of symptomatic hemolysis, and adverse events considered related to study drug (n = 4) were mild. The mean largest within-person percentage change in hemoglobin level between days 0 and 10 was −9.7% (95% confidence interval [CI], −13.5% to −5.90%) in G6PD-deficient adults receiving 0.50 mg/kg of SLD-PQ, −11.5% (95% CI, −16.1% to −6.96%) in G6PD-deficient boys aged 11–17 years, and −9.61% (95% CI, −7.59% to −13.9%) in G6PD-deficient boys aged 5–10 years. The lowest hemoglobin concentration at any point during the study was 92 g/L.

**Conclusion:**

SLD-PQ doses between 0.40 and 0.50 mg/kg were well tolerated in G6PD-deficient males in Mali.

**Clinical Trials Registration:**

NCT02535767.

## Teaser

This phase 1, open-label, nonrandomized, dose-adjustment trial showed that single doses of primaquine between 0.40 and 0.50 mg/kg were well tolerated in G6PD-deficient and G6PD-normal males in Mali, supporting the World Health Organization recommendation on the use of a single low of dose primaquine to reduce *Plasmodium falciparum* malaria transmission.

The success of malaria control and elimination programs supports global aspirations to eradicate human malaria in the coming decades [1]. To achieve these ambitions, new transmission-blocking tools and strategies are needed. In this context, the use of primaquine becomes important because it is the only commercially available drug that can stop the transmission of *Plasmodium falciparum* malaria from humans to anopheline mosquitoes.

Primaquine (PQ) use must be informed by safety considerations, as this drug causes dose-dependent hemolysis in individuals with enzymatic glucose-6-phosphate dehydrogenase (G6PD) deficiency, the most commonly inherited enzyme deficiency worldwide [2]. The safety of PQ depends primarily on its dose, as well as the variant of G6PD deficiency, which varies across geographic regions. In Africa, most G6PD deficiency variants are the mild, *A*− variant whilst in Asia, the variants are heterogeneous, with the Mediterranean variant being the most severe [3].

For treatment of *P. falciparum* gametocytes, the World Health Organization (WHO) recommends that a 0.25 mg/kg single low dose of PQ (SLD-PQ) be used without G6PD testing, in conjunction with standard artemisinin-based combination therapy, in areas approaching malaria elimination and/or facing drug resistance [4, 5]. This WHO recommendation is not backed by systematically collected evidence, and the WHO has expressed a need for additional clinical studies focused on the safety of SLD-PQ in G6PD-deficient (G6PD-d) individuals [5, 6].

Implementation of the WHO recommendation to use 0.25 mg/kg of SLD-PQ requires that each individual be weighed, PQ tablets be crushed and dissolved in water, and the corresponding amount of PQ solution for the 0.25 mg/kg dose be carefully measured. This process is labor intensive, introduces the possibility for dosing errors, and can be avoided by the establishment of age-based dosing bands. To develop age-based dosing bands, the therapeutic dose range of SLD-PQ, spanning the lowest efficacious dose to the highest safe dose in vulnerable individuals, must be established. This provides weight-based dose bands, which may then be converted to age-based dosing bands by using age-for-weight data [7]. This study aims to contribute to the establishment of the higher bound of the therapeutic dose range of SLD-PQ in African settings.

## METHODS

### Study Design and Participants

This study was an open-label, nonrandomized, dose-adjustment followed by age de-escalation trial of the safety of single-dose PQ in G6PD-d and G6PD-normal (G6PD-n) males in Mali without microscopically detected malaria parasite infection. Participants were enrolled sequentially.

In part 1, we investigated 3 single doses of PQ in adults: the first was a prespecified dose of 0.40 mg/kg in 7 individuals confirmed to be G6PD deficient according to a Carestart G6PD rapid diagnostic test (Access Bio, Somerset, NJ); this dose was demonstrated to be safe in a previous study [8]. The remaining 2 dose groups were determined by a data safety and monitoring board (DSMB), on review of data on adverse events (AEs) and hemoglobin (Hb) concentrations after follow-up of at least 10 days for each prior group. The dose was not escalated if any of the following occurred: ≥2 participants experienced acute hemolysis resulting in a Hb level drop of >30% or any participant experienced symptoms requiring blood transfusion or a serious AE (SAE), including acute renal failure and/or death, related to the study drug by day 10. The rationale for choosing a 30% drop was based on a prior study showing that Hb concentrations of <70 g/L were associated with increased mortality; our inclusion criteria ensured that starting Hb concentrations in participants were ≥100 g/L and thus that drops of <30% would not pose a mortality risk [9]). If none of these criteria were met, the DSMB selected the next dose, which could be escalated within a range of 0.05 to 0.2 mg/kg from the last study dose, not to exceed 0.75 mg/kg. This procedure continued until 3 dose groups were enrolled.

After enrollment of 3 dose groups, the DSMB reviewed study data and determined the highest dose of PQ tolerated in part 1, which was then administered to a control group of 7 G6PD-n individuals matched within 8 years of age to those enrolled in the group with the highest tolerable dose. Follow-up for the control group proceeded in the same manner as in the intervention groups. G6PD status was validated using semiquantitative spectrophotometry testing (with the OSMMR-D G-6-PD test; R&D Diagnostics, Aghia Paraskevi, Greece).

In part 2, we investigated the safety of single-dose PQ in G6PD-d boys at a dose 0.1 mg/kg lower than the highest safe dose found in adults to be conservative. For the first age group (11–17 years inclusive), we enrolled 7 G6PD-d boys and 7 G6PD-n boys (the control group). Following DSMB review of data by use of criteria described for part 1, if PQ was safe in this age group, a second age group (5–10 years inclusive) comprising 7 G6PD-d and 7 G6PD-n boys would be enrolled.

Participants were recruited from the town of Ouélessébougou and its surrounding villages in Mali by the Malaria Research and Training Centre of the University of Bamako, Mali. Prior to recruitment, the study team met with village leaders to arrange for information sessions on the trial for prospective participants in the community, seeking informed consent from those interested in participation. All individuals who provided consent were tested for G6PD deficiency, using the Carestart G6PD RDT test, and had their name, address, and phone number recorded. Those for whom tests revealed G6PD deficiency scheduled a date to visit the study clinic in Ouélessébougou for further screening, while those with a normal G6PD status revealed by testing were told they might be contacted at a later date for further screening.

Eligibility criteria included males, to reduce the possibility of incorrect classification of G6PD status by qualitative G6PD tests, which is known to occur more commonly in heterozygous females [10]; provision of written informed consent; ability to swallow oral medication; a Hb concentration of ≥100 g/L, as assessed by the HemoCue 301 analyzer (AB Leo Diagnostics, Helsingborg, Sweden); absence of malaria parasitemia according to a thick blood smear on enrollment; and agreement to abstain from the ingestion of grapefruit-containing products from 72 hours prior to the start of dosing until completion of follow-up. Eligible participants for part 1 of the study were aged 18–50 years and, for part 2 of the study, were enrolled on the basis of age group.

Participants who reported any of the following conditions were excluded: known positive results of human immunodeficiency virus (HIV) and/or hepatitis B virus tests, allergy to PQ, current receipt of treatment for tuberculosis or HIV infection or of any drugs that have hemolytic potential in G6PD-d individuals (including sulfonamides, dapsone, nitrofurantoin, nalidixic acid, ciprofloxacin, methylene blue, toluidine blue, phenazopyridine, and trimethoprim-sulfamethoxazole), use of antimalarial drugs ≤2 weeks before contact with the study team, blood transfusion of >500 mL within the last 3 months, high alcohol intake (>14 units of 10 g of alcohol per week) within the past 6 months, and/or reported use of illicit drugs (ie, marijuana, heroin, cocaine, and/or methamphetamine) ≤6 months before study entry. All eligible participants could be enrolled in the study one time, and any enrolled participant who vomited ≤1 hour after ingesting PQ was excluded from analysis.

The study was approved by the Ethics Committee of the Faculty of Medicine, Pharmacy, and Dentistry, University of Science, Techniques and Technologies of Bamako (approval no. 2015/89/CE/FMPOS), and by the Committee on Human Research at the University of California, San Francisco (institutional review board approval no. 14-14495). It also underwent human subjects review at the US Centers for Disease Control and Prevention and approved as non-engagement in human subjects research. The study was monitored by an external clinical trials monitor based in Bamako. Participants were compensated for time and travel. The trial was registered at Clinicaltrials.gov (registration no. NCT02535767).

### Procedures

After collection of day 0 samples, each participant received an oral dose of PQ according to his group assignment, after a fatty snack (biscuits) to minimize gastrointestinal symptoms. The study pharmacist prepared the dose by crushing a 15-mg tablet of PQ (Sanofi, Laval, Canada) in 15 mL of drinking water and administered the dose to the nearest 0.1 mL under direct observation [11].

Participants were evaluated at the study clinic at hours 1, 2, 4, 6, and 8 and days 1, 2, 3, 4, 5, 6, 7, 8, 9, 10, 14, and 28 following treatment. On all follow-up days, starting approximately 24 hours following PQ administration, a clinical examination was conducted, and blood samples were collected for measurement of the Hb concentration and for preparation of slides to determine malaria parasite and reticulocyte counts (reticulocytosis was defined as a reticulocyte count of >2.0% of the total red blood cell count). To assess for oxidative stress, a noninvasive methemoglobin measurement was performed at all follow-up time points, using a Masimo Rad-57 pulse oximeter (Masimo, Irvine, CA; methemoglobinemia was defined as a methemoglobin level of >3% of the total Hb level). Owing to technical issues, the pulse oximeter was not functional until the start of enrollment of the second dose group. Urine samples were also collected on each day of follow-up, to assess for hemolysis by using a urine color chart developed by Hillmen and Hall [12]. If a ≥30% drop in baseline Hb levels was observed by day 7 of follow-up, a blood sample was collected on day 10 for additional assessment of serum bilirubin, urea, and creatinine levels.

AEs were assessed actively during all follow-up visits and passively through the availability of study clinicians 24 hours/day, 7 days/week. We recorded the duration and severity of AEs (mild, grade 1; moderate, grade 2; severe, grade 3; and life threatening, grade 4); relationship to the study drug, according to the study physician(s) (definite, probable, possible, remotely possible, unrelated, or unclassifiable); actions taken; and outcome. AEs were further assessed as serious or not serious, according to the treating physician. SAEs were prespecified in the study protocol and adapted by the DSMB prior to trial initiation and were defined as any of the following: development of clinical signs or symptoms of distress, including any requirement for hemodialysis; laboratory values indicating severe hemolytic anemia, including a drop in the Hb level of >40% from baseline levels; a need for blood transfusion; the development of hemoglobinuria, identified by black coloration in the urine associated with a rise in creatinine level; and any SAE, defined as an untoward medical occurrence or effect that, at any dose, results in death, is life threatening, requires hospitalization, or results in persistent or significant disability or incapacity that is clearly, probably, or possibly related to the study drug.

G6PD testing was conducted using 3 methods: qualitative phenotypic testing, semiquantitative testing, and genotyping. Qualitative phenotypic rapid testing using the Carestart G6PD test was conducted as a screening test throughout the study. Additionally, the field team conducted additional qualitative OSMMR-D G-6-PD tests in part 1 after enrollment of the first 3 individuals, excluding individuals with discordant results between the 2 qualitative tests [13]. Semiquantitative spectrophotometry testing was conducted on all individuals, using cryopreserved blood samples (0.1 mL of whole blood) collected prior to PQ administration, and results were used to determine the final G6PD status for participants, for inclusion in analysis. Genotypic testing was conducted for detection of the G6PD G202A single-nucleotide polymorphism (SNP) in all individuals, in addition to the A376G SNP in adults; the *A* variant of both SNPs was known to be common among G6PD-d individuals in Africa at the time of study design [14]. G6PD-d individuals with any SNPs that were not assessed, including 986C, which was later identified to be common in Mali, were identified as wild type in this study [15].

Cytochrome P450 2D6 SNPs were identified for adults, and polymerase chain reaction (PCR) methods were used to detect malaria parasites in all participants, using dried blood spots collected on filter paper prior to PQ administration (described in Supplemental Material).

### Outcomes

The primary safety outcome was the largest within-person percentage decrease in Hb concentration between baseline levels (day 0) and day 10 following PQ administration. Prespecified secondary outcomes consisted of the incidence of AEs, graded by severity and relation to the study drug; the occurrence of acute hemolytic anemia at each PQ dose, including absolute and fractional changes in Hb concentration on day 7 and day 28 as compared to baseline; urine color assessment; reticulocyte count; total and direct bilirubin levels if a within-person Hb concentration drop of ≥30% from baseline levels was seen in an individual between day 0 and 7 (inclusive); methemoglobin concentration and development of physical signs or symptoms of hemolytic anemia; comparison of the change in the Hb concentration, the frequency and severity of AEs, and the occurrence of markers of acute hemolytic anemia between G6PD-d and G6PD-n participants; and exploratory studies among adults on whether cytochrome P450 2D6 (*CYP2D6*) SNPs are associated with hemolysis in G6PD-d individuals (Supplementary Materials).

### Statistical Analysis

Based on preliminary data, sample size calculations assumed that the average Hb concentration before treatment would be 125 g/L and that the standard deviation for the within-person change in Hb concentration after treatment would be 17 g/L. Our sample size calculation enabled detection of a ≥15% within-person drop in Hb concentration after treatment, compared with the null hypothesis of no drop, with 80% power and a 1-tailed significance level of 0.05 and requiring 7 individuals per group. A sample of this size also enabled the detection of a within-person drop in Hb concentration of ≥20% among the G6PD-d participants as compared to G6PD-n controls.

For the analysis of changes in Hb concentrations, the outcomes were the mean largest within-person percentage decrease in Hb concentration between baseline and day 7, day 10, and day 28 after PQ treatment. Within age groups (age 5–10, 11–17, and ≥18 years), we used a regression model that adjusted for baseline Hb concentration, to determine whether there was a significant difference in the outcomes between G6PD-d and G6PD-n participants after treatment.

AEs were summarized descriptively for each dosing group, using frequencies of observed events, severity, and proportion of participants experiencing at least 1 event.

All analyses were conducted using Stata v12 (StataCorp, College Station, TX).

## RESULTS

**Figure 1.**
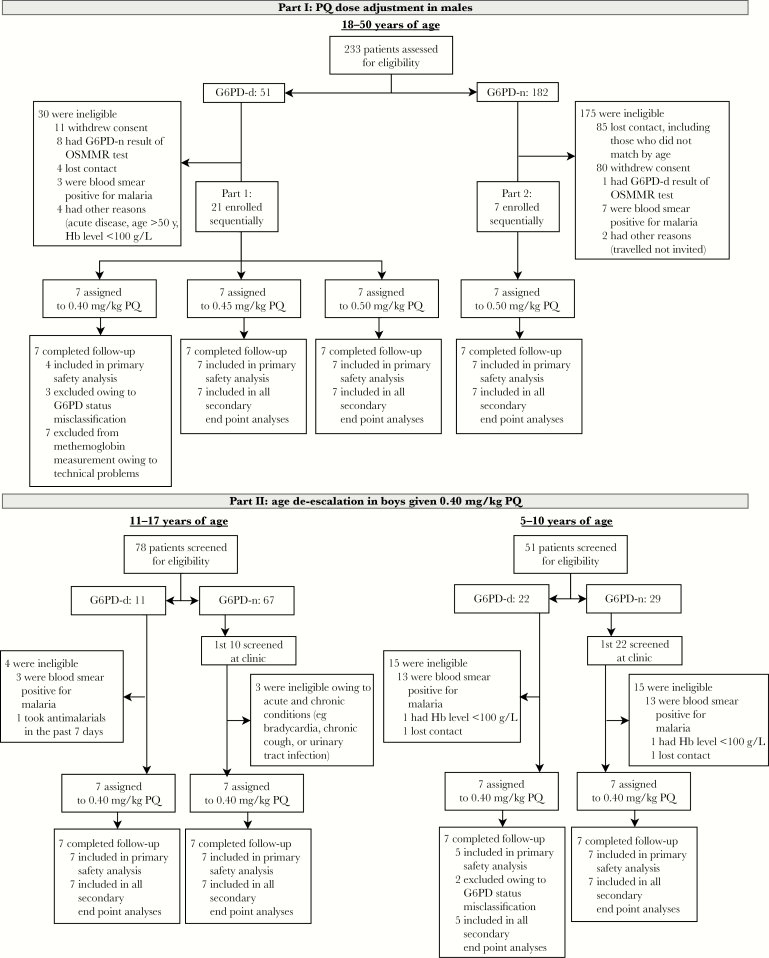
Trial profile. Primaquine (PQ) was given as a single dose on the day of enrollment. All enrolled individuals completed the study; none were lost to follow-up. G6PD-d, glucose-6-phosphate dehydrogenase deficient; G6PD-n, glucose-6-phosphate dehydrogenase normal. Hb, hemoglobin.

In part 1, 28 adult males were enrolled between 13 August and 19 December 2015 (Figure 1). The dose groups of PQ were 0.40 mg/kg, 0.45 mg/kg, and 0.50 mg/kg among G6PD-d males and 0.50 mg/kg in the control group of G6PD-n males (Table 1). The first 3 individuals enrolled in the 0.40 mg/kg group were incorrectly classified (by the Carestart screening test) as G6PD-d and were G6PD-n according to semiquantitative spectrophotometry (all were excluded from analyses; Supplementary Table 1). All G6PD-n individuals were wild type for both the G6PD G202A and A376G allele, most (15 of 18) G6PD-d individuals had SNPs for the G202A and A376G allele and had partial G6PD deficiency, and 3 of 18 G6PD-d individuals were identified as wild type but had total G6PD deficiency according to semiquantitative spectrophotometry.

**Table 1.** Baseline Characteristics of Participants Overall and by Primaquine (PQ) Treatment Group

Characteristic	Overall (n = 25)	G6PD-d Group, by PQ Dose			G6PD-n Control Group^a^ (n = 7)
		0.40 mg/kg (n = 4)	0.45 mg/kg (n = 7)	0.50 mg/kg (n = 7)	
Part 1					
Age, y	35 (18–50)	29 (18–50)	35 (26–50)	37 (25–47)	37 (28–43)
Weight, kg	63.0 (44.2–83.0)	55.8 (44.2–70.1)	60.5 (52–74.4)	62.9 (56.8–68.5)	69.9 (59.9–83.0)
Baseline Hb level, g/L	149 (120–175)	138 (120–151)	152 (144–175)	151 (136–162)	150 (136–165)
		Age 11–17 y		Age 5–10 y	
	Overall (n = 26)	G6PD-d Group (n = 7)	G6PD-n Group (n = 7)	G6PD-d Group (n = 5)	G6PD-n Group (n = 7)
Part 2					
Age, y	10 (5–17)	13 (11–16)	13 (11–17)	7 (5–8)	7 (5–9)
Weight, kg	28 (16–48)	35 (29–44)	35 (25–48)	20 (17–24)	23 (18–29)
Baseline Hb level, g/L	123 (102–145)	127 (121–130)	130 (116–142)	117 (108–142)	119 (102–145)

Data are mean value (range). Part 1 was a PQ dose de-escalation trial in men (age ≥18 years). Part 2 was an age de-escalation trial in boys given 0.40 mg/kg PQ.

Abbreviations: G6PD-d, glucose-6-phosphate dehydrogenase deficient; G6PD-n, glucose-6-phosphate dehydrogenase normal; Hb, hemoglobin.

^a^Received 0.50 mg/kg PQ.

In part 2, 28 boys were enrolled between 12 May 2016 and 10 January 2017 (Figure 1). Two boys in the G6PD-d group aged 5–10 years were misclassified by the Carestart screening test and were G6PD-n according to semiquantitative spectrophotometry (all were excluded from analyses; Supplementary Table 3). All G6PD-n boys enrolled were wild type at the G202A allele. Among G6PD-d boys, 7 of 12 had SNPs at G202A, and 5 of 12 were identified as wild type, of whom 4 were characterized with partial G6PD deficiency and 1 was characterized with total G6PD deficiency, using semiquantitative testing.

Among men (ie, participants in part 1), the mean largest within-person percentage change in Hb concentration among G6PD-d individuals receiving the highest tolerable dose of PQ (0.50 mg/kg) was −9.7% (95% CI, −13.5% to −5.90%), whereas their G6PD-n counterparts experienced a change of −7.89% (95% CI, −11.4% to −4.37%; *P* = .320; Table 2 and Supplementary Figure 1). Among boys aged 5–10 years (who were in part 2), the mean largest within-person percentage change in Hb concentration among G6PD-d participants was −9.61% (95% CI, −3.08% to −16.1%), whereas their G6PD-n counterparts experienced a change of −5.24% (95% CI, −.252% to −10.2%; *P* = .199). Similarly, among boys aged 11–17 years (who were also in part 2), the mean largest within-person percentage change in Hb concentration among G6PD-d participants was −11.5% (95% CI, −6.96% to −16.1%), whereas their G6PD-n counterparts experienced a change of −6.89% (95% CI, −2.46% to −11.3%; *P* = .072). There was no significant difference between the mean largest within-person percentage change in Hb concentration among G6PD-d adult men (n = 18; −10.5%; 95% CI, −13.3% to −7.6%) and that among G6PD-d boys aged 5–17 years (n = 12; −10.7%; 95% CI, −13.9% to −7.59%; *P* = .90).

**Table 2.** Within-Person Percentage Changes and Day of Largest Change in Hemoglobin (Hb) Concentrations, by Primaquine (PQ) Treatment Group

Treatment Group	Largest Change Between Days 0 and 10		Change From Days 0 to 7		Change From Days 0 to 28		Mean Follow-up Day of Largest Change, Mean (95% CI)
	Percentage (95% CI)^a^	*P* ^b^	Percentage (95% CI)^a^	*P* ^b^	Percentage (95% CI)^a^	*P* ^b^	
Part 1							
G6PD-n control group^c^ (n = 7)	−7.89 (−11.4 to −4.37)		−2.13 (−8.10–3.84)		−2.49 (−6.88–1.91)		7.9 (5.0–10.8)
G6PD-d group, by PQ dose							
0.40 mg/kg (n = 4)	−16.8 (−24.7 to −8.93)	<.001	−7.53 (−19.2–4.11)	.009	−1.00 (−19.4–17.4)	.215	7.0 (3.6–10.4)
0.45 mg/kg (n = 7)	−7.63 (−12.5 to −2.75)	.662	−4.62 (−10.9–1.68)	.587	−2.33 (−8.99–4.32)	.638	9.4 (1.6–17.2)
0.50 mg/kg (n = 7)	−9.72 (−13.5 to −5.90)	.320	−3.77 (−9.77–2.23)	.606	−0.754 (−6.67–5.16)	.481	7.7 (3.9–11.5)
Part 2							
Age 11–17 y							
G6PD-n group (n = 7)	−6.86 (−11.3 to −2.46)		0.314 (−3.69–4.31)		−0.662 (−5.23–3.90)		9.0 (.9–17.1)
G6PD-d group (n = 7)	−11.5 (−16.1 to −6.96)		−6.89 (−10.5 to −3.30)	.006	−6.59 (−13.5–.300)	.111	13.4 (4.0–22.8)
Age 5–10 y							
G6PD-n group (n = 7)	−5.24 (−10.2 to −.252)		4.46 (−3.01–11.9)		1.89 (−6.62–10.4)		7.6 (−1.8–16.9)
G6PD-d group (n = 5)	−9.61 (−16.1 to −3.08)	.199	0.017 (−13.2–13.3)	.378	−1.02 (−11.9–9.89)	.407	11.4 (4.1–18.7)

Part 1 was a PQ dose de-escalation trial in men (age ≥18 years). Part 2 was an age de-escalation trial in boys given 0.40 mg/kg PQ.

Abbreviations: CI, confidence interval; G6PD-d, glucose-6-phosphate dehydrogenase deficient; G6PD-n, glucose-6-phosphate dehydrogenase normal; Hb, hemoglobin.

^a^Negative values indicate drops in the Hb level.

^b^Adjusted for baseline Hb level. Within age groups, we used a regression model that adjusted for baseline Hb level to compare G6PD-d and G6PD-n participants.

^c^Received 0.50 mg/kg PQ.

The mean largest percentage change in Hb concentration was a 16.8% decrease between day 0 and day 10, which occurred in the group of men who received 0.40 mg/kg of SLD-PQ. This group included the individual with the largest within-person percentage change in Hb concentration (−23%; this person had the *A* variant of G6PD deficiency); this man had a Hb concentration of 147 g/L at baseline and 113 g/L 9 days following PQ administration, was positive for *P. falciparum* malaria at baseline by PCR, was positive by blood smear on days 1 and 3–7, and received a symptomatic malaria diagnosis on days 6–9 following treatment with PQ. At day 28, this participant’s Hb concentration was 135 g/L. For all participants enrolled, the lowest absolute Hb concentration was 92 g/L, observed in a 6-year-old G6PD-d boy; he had a Hb concentration of 112 g/L at baseline, the nadir Hb concentration on day 10, no malaria parasites detected by blood smear, and a Hb concentration of 106 g/L by day 28.

**Figure 2.**
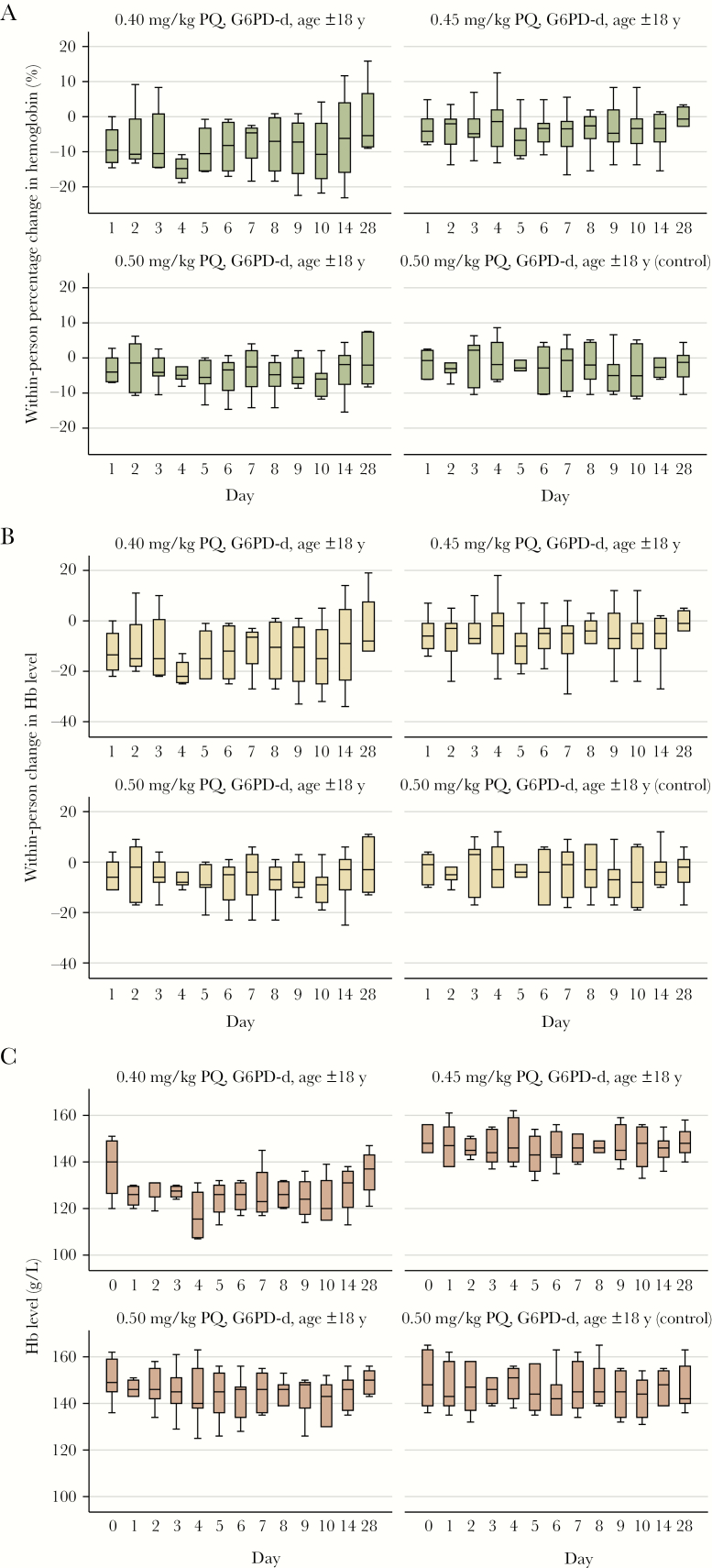
Hemoglobin (Hb) concentration over 28 days of follow-up, by primaquine (PQ) dose group, among individuals in part 1 of the study. *A*, Within-person percentage change from baseline. *B*, Within-person change from baseline concentration. *C*, Absolute concentration. Boxplots denote medians (lines within boxes), interquartile ranges (upper and lower limits of boxes), and ranges (whiskers). G6PD-d, glucose-6-phosphate dehydrogenase deficient.

**Figure 3.**
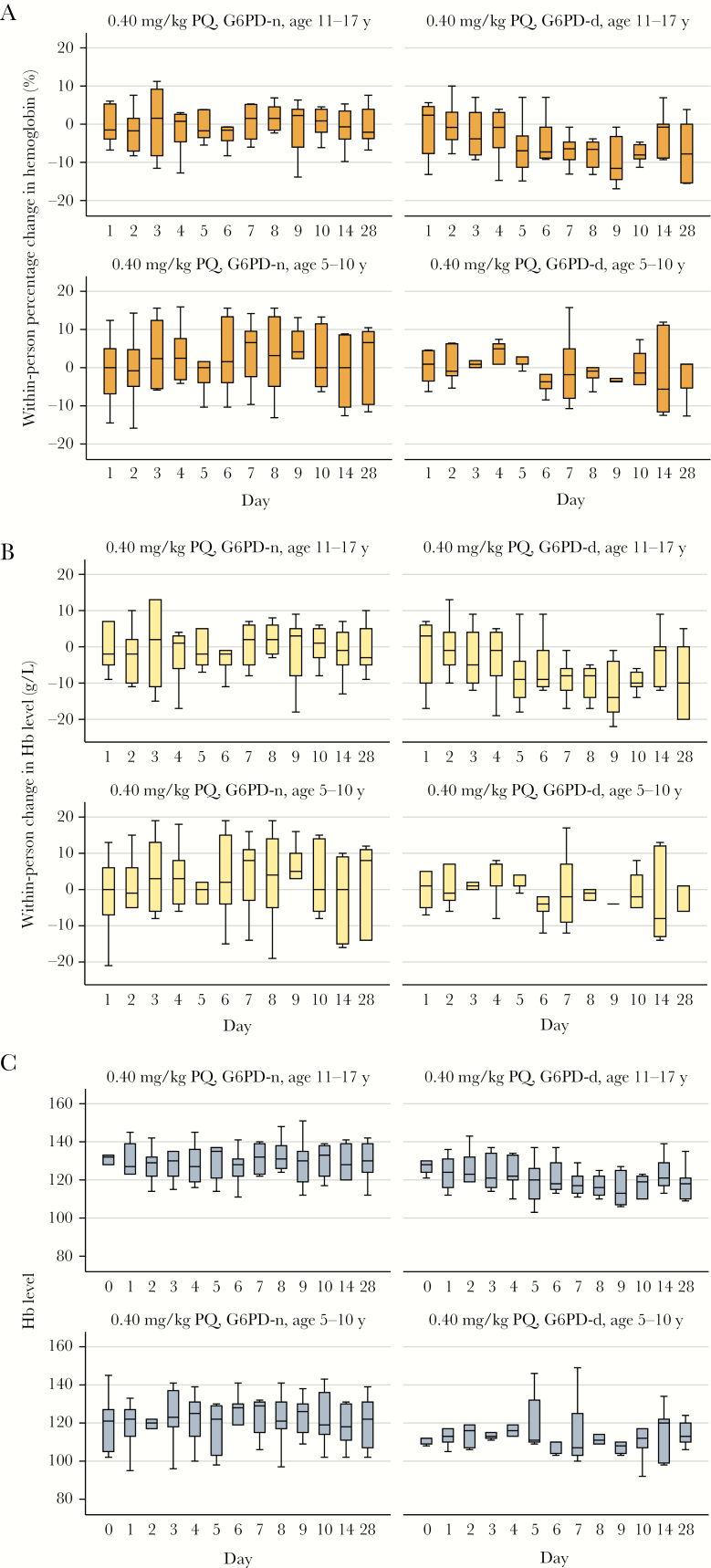
Hemoglobin (Hb) concentration over 28 days of follow-up, by enrollment group (G6PD status and age), among individuals in part 2 of the study. *A*, Within-person percentage change from baseline. *B*, Within-person change from baseline concentration. *C*, Absolute concentration. Boxplots denote medians (lines within boxes), interquartile ranges (upper and lower limits of boxes), and ranges (whiskers). G6PD-d, glucose-6-phosphate dehydrogenase deficient; G6PD-n, glucose-6-phosphate dehydrogenase normal.

During the 28 days of follow-up, no participants experienced a ≥30% drop in Hb level (Figures 2 and 3). There were no events of acute hemolysis, SAEs, or symptoms or signs of acute renal failure, hemolytic anemia, hemoglobinuria, or methemoglobinemia observed. A total of 24 participants (47%) had an AE during follow-up; most AEs (44 of 46 [96%]) were mild (grade 1), of which 4 were considered related to PQ (Table 3). All AEs resolved during study follow-up, and none resulted in stopping participation. Thirteen participants (24%) had reticulocytosis, of whom 12 were G6PD deficient (Supplementary Figure 2).

**Table 3.** Adverse Events (AEs) Among Study Participants, by Primaquine (PQ) Treatment Group

Variable	G6PD-d Group, by PQ Dose			
	0.40 mg/kg (n = 4)	0.45 mg/kg (n = 7)	0.50 mg/kg (n = 7)	G6PD-n Control Group^a^ (n = 7)
Part 1				
Participants with AE	4 (100)	4 (57)	2 (29)	2 (29)
Any AE	4	8	2	2
Drug-related AE	0	0	0	0
AE type				
Cough, mild	0	3	0	0
Fever, mild	1	0	0	0
Headache, mild	1	1	0	1
Headache, moderate	0	0	1	0
Insect bites, mild	0	1	0	0
Rhinobronchitis, mild	0	1	0	1
Skin boil, mild	0	0	1	0
Symptomatic malaria, mild	2	2	0	0
	Age 11–17 y		Age 5–10 y	
	G6PD-d Group	G6PD-n Group	G6PD-d Group	G6PD-n Group
Part 2				
Participants with AE	3 (43)	1 (14)	2 (40)	6 (86)
Any AE	4	1	8	18
Drug-related AE	1	0	1	2
AE type				
Abdominal pain, mild	0	0	2	6
Adenopathy, mild	0	0	0	1
Back pain, mild	0	0	1	0
Conjunctivitis, mild	0	1	0	0
Dental pain, mild	0	0	0	1
Diarrhea, mild	1	0	0	0
Ear infection, mild	0	0	0	1
Fever, mild	1	0	0	1
Headache, mild	1	0	0	1
Injury in big toe, mild	0	0	0	2
Irregular heartbeat, mild	0	0	0	1
Loss of appetite, mild	0	0	1	0
Nasopharyngitis, mild	0	0	0	3
Runny nose, mild	0	0	1	0
Skin boil, moderate	1	0	0	0
Sore throat, mild	0	0	0	1
Vomiting, mild	0	0	3	0

Data are no. (%) or no. of participants. Part 1 was a PQ dose de-escalation trial in men (age ≥18 years). Part 2 was an age de-escalation trial in boys given 0.40 mg/kg PQ. There were no severe or serious AEs. There were no AEs that caused stopping rules. All events were self-reported. Mild events were defined as those causing no or minimal interference with usual social and functional activities. Moderate events were defined as those causing greater than minimal interference. Severe events were defined as those causing an inability to perform usual social and functional activities.

Abbreviations: G6PD-d, glucose-6-phosphate dehydrogenase deficient; G6PD-n, glucose-6-phosphate dehydrogenase normal.

^a^Received 0.50 mg/kg PQ.

At the screening visit, although no participants were symptomatic for malaria and all were smear negative, PCR analysis of stored blood samples revealed that 41% participants (21 of 51) had asymptomatic malaria parasitemia. During follow-up, 14 participants tested positive for malaria parasites on blood smear, with 4 being symptomatic (Supplementary Table 4). There was no evidence that the within-person change in Hb concentration during follow-up was associated with malaria status (Supplementary Figure 3).

## DISCUSSION

This study found that a single dose of PQ as high as 0.50 mg/kg was well tolerated among G6PD-d adults and that a 0.40-mg/kg dose was well tolerated in G6PD-d adults and children. Our study is the first to systematically investigate the safety of SLD-PQ at doses of ≥0.40 mg/kg in G6PD-d individuals without malaria, addressing the evidence gap identified by the WHO Expert Review Group that issued the recommendation to use SLD-PQ. Despite consistent measures of reduced Hb concentrations in G6PD-d participants exposed to SLD-PQ, compared with G6PD-n participants, the reductions in Hb levels were small and not clinically relevant.

This study also provides data to define the therapeutic dose range of SLD-PQ in West African settings, which will support field implementation of the WHO recommendation, currently based on patient weight and challenged by a requirement to dissolve tablets and carefully measure the correct amount of solution to give to the patient [6, 7]. Age-based dose bands for SLD-PQ for individuals in African settings were recently modeled by Hayes et al [16], who observed a need for clinical studies to ascertain the therapeutic dose range and preliminarily assumed this to be between 0.1 and 0.4 mg/kg. The lower bound of the therapeutic dose range of SLD-PQ is between 0.125 and 0.25 mg/kg, and our study suggests that the upper bound may be extended to 0.40 mg/kg in West African settings [17–20]. As the severity of G6PD deficiency can be approximately characterized by geography, these results should not be applied to Asian settings.

Other studies on SLD-PQ to date have focused on the safety of the WHO-recommended dose of 0.25 mg/kg of SLD-PQ, which was demonstrated to be safe in Senegal, when given with artemether-lumefantrine, dihydroartemisinin-piperaquine, or artesunate-amodiaquine to 54 phenotypically G6PD-d patients with malaria [21]; in Tanzania, when given with artemether-lumefantrine to 33 phenotypically G6PD-d patients with malaria [22]; and at the Thai-Myanmar border, when given with dihydroartemisinin-piperaquine to 124 phenotypically G6PD-d individuals in a mass drug administration campaign [23]. A study investigating the safety of 0.25 and 0.40 mg/kg PQ in asymptomatic G6PD-d patients with malaria in Burkina Faso has been completed [24], although results have not yet been reported.

As malaria parasite infection can reduce hemoglobin concentration, this study was intended to examine the isolated effect of PQ on individuals without malaria. However, many individuals enrolled had asymptomatic malaria (40% during screening and 26% during follow-up), of whom 4 (8%) presented with symptomatic malaria on follow-up. While it is not possible to determine whether Hb level drops in these individuals were due to malaria parasite infection or to SLD-PQ, the results suggest that SLD-PQ is well tolerated in G6PD-d individuals with and those without malaria.

The main limitations of this study include a small sample size, the exclusion of women and, as an ethical precaution taken to ensure the safety of study participants, and vulnerable individuals who were already anemic on enrollment. As SLD-PQ is now being rolled out in various countries in Africa, further assessment of its safety should be performed using pharmacovigilance mechanisms, with an emphasis on vulnerable individuals who are anemic, have HIV infection and/or tuberculosis, and/or are unaware of their pregnancy when they take PQ.

In summary, these results provide evidence for extending the upper bound of the therapeutic dose range of SLD-PQ to 0.40 mg/kg in sub-Saharan Africa. This evidence may be used to facilitate the age-based dosing of SLD-PQ in sub-Saharan Africa.

## Supplementary Data

Supplementary materials are available at *The Journal of Infectious Diseases* online. Consisting of data provided by the authors to benefit the reader, the posted materials are not copyedited and are the sole responsibility of the authors, so questions or comments should be addressed to the corresponding author.

Supplementary Material

## Notes

***Acknowledgments.*** We thank the DSMB (Prof Daniel Chandramohan [chair], Prof Ric Price, Dr Joel Tarning, and Dr Sophie Zaloumis); the laboratory and field staff, for their hard work; the participants and members of the community health association in Ouelessebougou, for their cooperation throughout the study; and Sanofi, for their kind donation of PQ.

***Financial support.*** This work was supported by the Bill and Melinda Gates Foundation (OPP1089413, OPP1089413 [to A.D.], and AFIRM OPP1034789 [to T. B.]), the Government of Mali (to A. D.), the European Research Council (fellowship ERC-2014-StG 639776 to T. B.), and the President’s Malaria Initiative (to J. H.).

***Potential conflicts of interest.*** All authors: No reported conflicts of interest. All authors have submitted the ICMJE Form for Disclosure of Potential Conflicts of Interest. Conflicts that the editors consider relevant to the content of the manuscript have been disclosed.
