# Erratum

**DOI:** 10.1093/infdis/jiy074

**Published:** 2018-03-08

“Safety of single dose primaquine in G6PD-deficient and G6PD-normal males in Mali without malaria: an open- label, phase 1, dose-adjustment trial” by Chen et al. [J Infect Dis 2018; doi:10.1093/infdis/jiy014]. This is an Open Access article and should have contained the following copyright line: © The Author(s) 2018. Published by Oxford University Press for the Infectious Diseases Society of America. This is an Open Access article distributed under the terms of the Creative Commons Attribution License (http://creativecommons.org/licenses/by/4.0/), which permits unrestricted reuse, distribution, and reproduction in any medium, provided the original work is properly cited.

The authors regret the error.

