# Supplementary material for: Safety of Single-Dose Primaquine in G6PD-Deficient and G6PD-Normal Males in Mali Without Malaria: An Open-Label, Phase 1, Dose-Adjustment Trial
Source: J Infect Dis. 2018 Jan 12;217(8):1298–308. doi: 10.1093/infdis/jiy014 (PMC5974787; doi:10.1093/infdis/jiy014)
Supplement: Supplementary Material [file jiy014_suppl_supplementary_material.docx]

**Supporting Information: Safety of single dose primaquine in G6PD-deficient males in Mali without malaria: an open-label phase 1 dose-adjustment trial**

Ingrid Chen, Halimatou Diawara, Almahamoudou Mahamar, Koualy Sanogo, Sekouba Keita, Daouda Kone, Kalifa Diarra, Moussa Djimde, Mohamed Keita, Joelle Brown, Michelle E. Roh, Jimee Hwang, Helmi Pett, Maxwell Murphy, Mikko Niemi, Bryan Greenhouse, Teun Bousema, Roly Gosling, Alassane Dicko

Contents:

1. Quality checks for hemocue measurements: page 1
2. Absolute hemoglobin concentration (g/L) over 28 days of follow-up by individual in each primaquine dose group: page 2
3. Detailed methods for genotyping and molecular detection of malaria parasites (PCR): page 3
4. Reticulocyte data: page 4
5. G6PD testing and CYP 2D6 genotyping results: pages 5 – 7
6. Malaria infection detected by PCR at baseline, and by blood smear and symptoms on follow up: page 8
7. Sensitivity analyses investigating potential correlation between malaria infection and within-person changes in hemoglobin concentration during follow-up: page 9
8. References: page 9
9. Quality checks for hemocue measurements

To ensure the accuracy of hemoglobin concentration measurements using the Hemocue, if the difference between the value of the Hb concentration that day and the previous measure was ≥1.5 g/dL, a second measurement was taken. If the second measurement was not different (< 1.5 g/dL) from first measurement, the first was recorded. If it was different (≥1.5 g/dL), a third measurement was performed. If the third measurement was not different (< 1.5 g/dL) from the second, the second was recorded. If the third was different (≥1.5 g/dL) from the second, a complete blood count (CBC) was performed to confirm and the closest measure to the CBC was recorded.

1. Absolute hemoglobin concentration (g/L) over 28 days of follow-up by individual in each primaquine dose group

Figure S3.


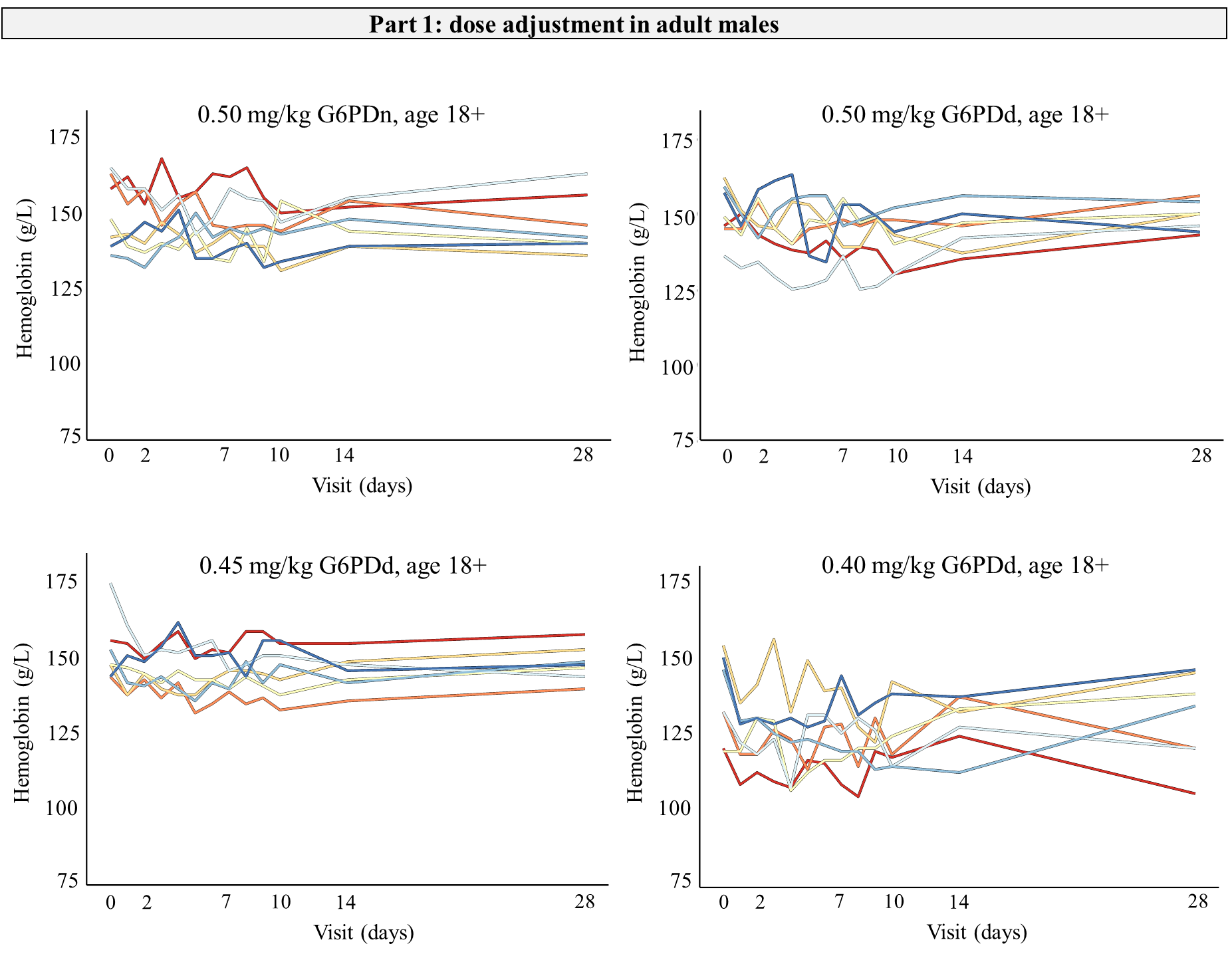


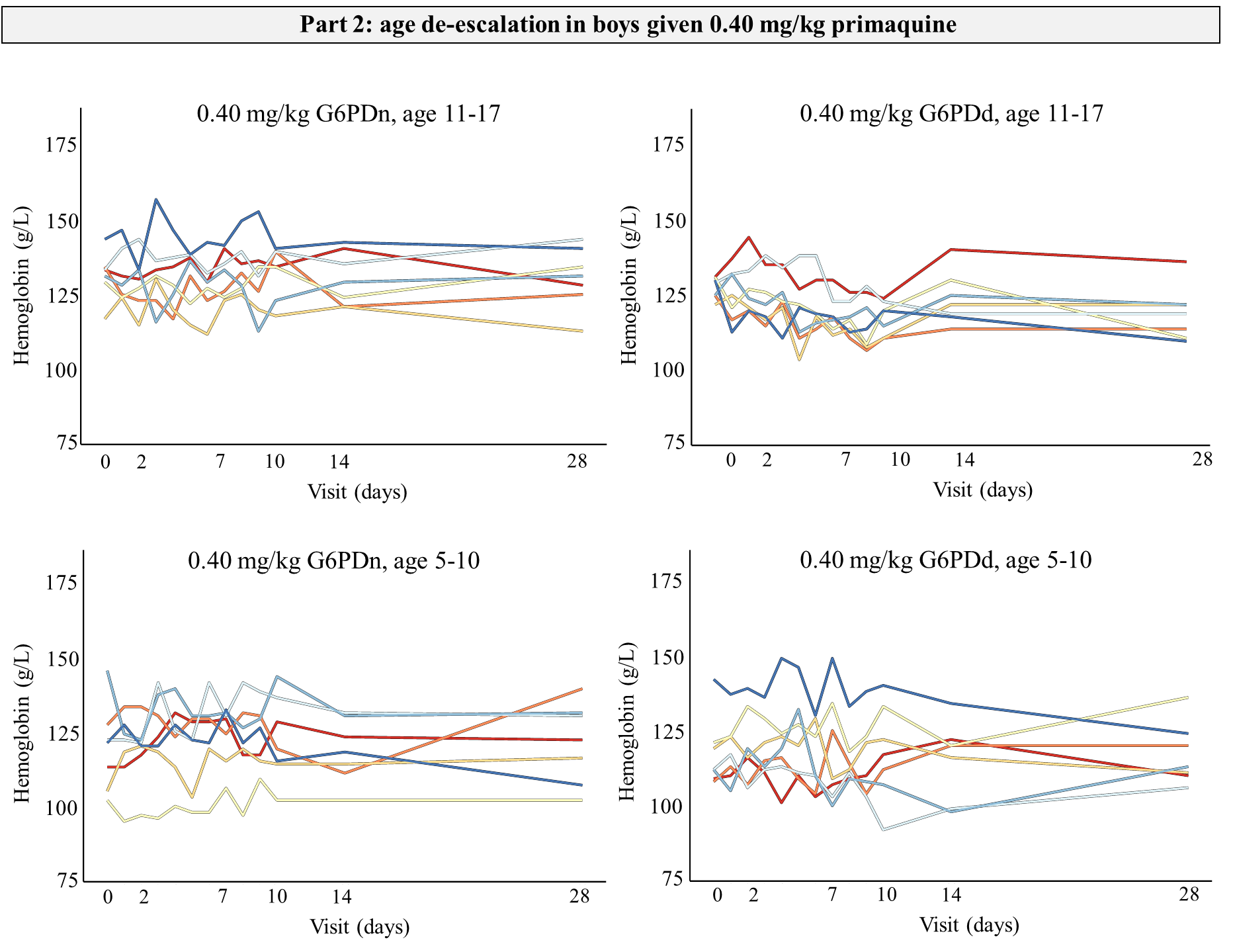


1. Detailed methods for genotyping and molecular detection of malaria parasites (PCR)

G6PD and Cytochrome P450 2D6 (CYP2D6) genotyping was conducted on genomic DNA extracted from a cryopreserved blood sample (1.0 mL of whole blood in EDTA) collected from each participant prior to primaquine administration.

In part 1 of the study, adults were genotyped for CYP2D6 *2, *3, *4, *6, *7, *8, *9, *10, *11, *17, *18, *19, *20, *29, *40, and *41 alleles and G6PD G202A and A376G SNPs, known to be unique to the G6PD A- variant seen in 90% of G6PD-deficient individuals in Africa,[^1^](#_ENREF_1) using OpenArray technology on a QuantStudio 12K Flex real-time PCR system (Life Technologies, Carlsbad, CA, USA). The OpenArray was repeated with and without preamplification, after which results for *29 were excluded due to inconsistencies. *29-assay was re-done in single assay format and incorporated into final results. The CYP2D6 copy number was determined with a TaqMan copy number assay targeting exon 9 on the QuantStudio 12K Flex system, and CYP2D6 metabolizer status was inferred from the genotypes using the Activity Score (AS) method.[^2^](#_ENREF_2) Division into the four metabolizer phenotypes (poor, intermediate, extensive and ultrarapid metabolizers) was done based on the methods previously described, where an AS of 1.0 corresponds to an intermediate metabolizer phenotype.[^3^](#_ENREF_3)

In part 2 of the study, children were genotyped for G6PD using restriction fragment length polymorphism for the G6PD A- G202A allele. PCR was performed using methods as previously described.[^4^](#_ENREF_4) CYP2D6 genotyping was not performed but may be undertaken at a later date.

Molecular detection of malaria parasites was conducted using Polymerase Chain Reaction (PCR) methods from dried blood spots on filter paper for samples from all participants collected prior to primaquine administration. DNA was extracted from dried blood spots using the saponin/Chelex method.[^5^](#_ENREF_5) Nested PCR for cytochrome B mitochondrial DNA was conducted following previously published methods and amplification product was detected by agarose gel electrophoresis.[^6^](#_ENREF_6) All PCR was performed on a Bio-Rad Thermocycler T100 (Bio-Rad Laboratories, USA). Following PCR amplification, samples positive by cytochrome B PCR had their amplification product digested using AluI restriction enzyme (New England BioLabs, USA) and were detected by agarose gel electrophoresis to determine species.[^7^](#_ENREF_7)

1. Reticulocyte data

Reticulocyte count ranged from 0.5% to 2.9% in adults and 0.5% and 4.9% in children 5-17 years of age. Among G6PD-d adults in part 1, 6 (24%) had reticulocytosis (defined as reticulocyte >2%), including one participant in the 0.50 mg/kg group, three in the 0.45 mg/kg group, and two in the 0.40 mg/kg group. No adults in the 0.50 mg/kg, G6PD-n control group experienced reticulocytosis. Among children in the phase 2 group, 7 (27%) had reticulocytosis; 1 G6PD-n child 5-10 years of age, 4 G6PD-d children 11-17 years of age, and 2 G6PD-d children 5-10 years of age. None of the G6PD-n children 11-17 years of age experienced reticulocytosis.

Figure S2. Reticulocyte count (%) over 28 days of follow-up, by primaquine treatment group

Boxplot key: median (line) IQR (box), and range (whisker)


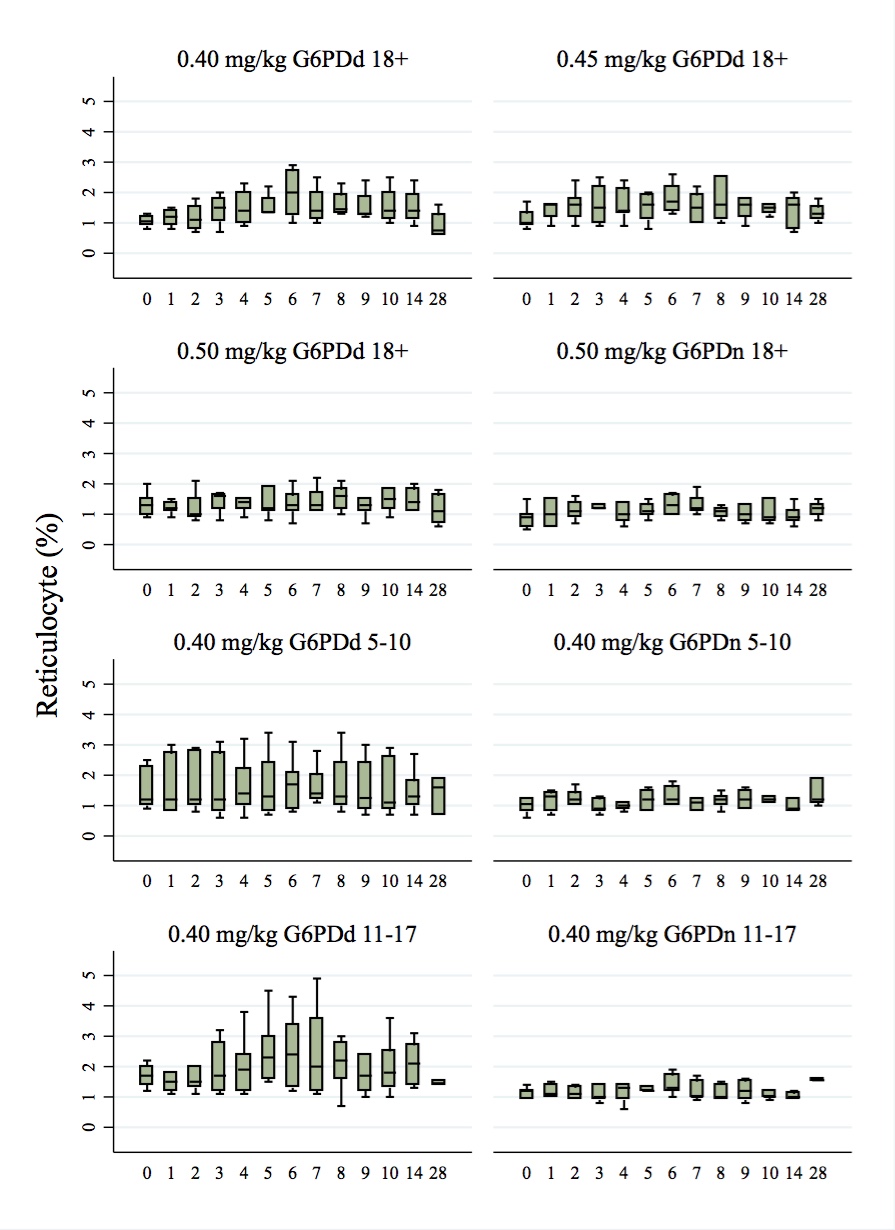


1. G6PD testing and CYP 2D6 genotyping results

In part 1, all participants with SNPs 202A and 376G on genotyping tested as partially G6PD-d (intermediate deficiency) using semi-quantitative assessment. Three participants (#9, #14, and #20), were classified as totally G6PD-d according to semi-quantitative testing, but did not have SNPs 202A or 376G during genotyping.

Table S1. Part 1 Results of G6PD testing and CYP 2D6 genotyping by individual participant

| Group / Participant ID# | | G6PD testing^1^ | | | | CYP 2D6 metabolism^2,3^ |
| --- | --- | --- | --- | --- | --- | --- |
|  |  | Qualitative (screening) | | Semi-quantitative | Genotyping (SNPs 202A and 376G) |  |
|  |  | Carestart | OSMMR |  |  |  |
| 0.40 mg/kg | 1 | G6PD-d | Test not conducted | G6PD-n | WT | EM |
|  | 2 | G6PD-d |  | G6PD-n | WT | PM |
|  | 3 | G6PD-d |  | G6PD-n | WT | IM |
|  | 4 | G6PD-d | G6PD-d | Partially deficient | A- | IM |
|  | 5 | G6PD-d | G6PD-d | Partially deficient | A- | EM |
|  | 6 | G6PD-d | G6PD-d | Partially deficient | A- | EM |
|  | 7 | G6PD-d | G6PD-d | Partially deficient | A- | EM |
| 0.45 mg/kg | 8 | G6PD-d | G6PD-d | Partially deficient | A- | EM |
|  | 9 | G6PD-d | G6PD-d | Totally deficient | WT | EM |
|  | 10 | G6PD-d | G6PD-d | No data | A- | IM |
|  | 11 | G6PD-d | G6PD-d | Partially deficient | A- | EM |
|  | 12 | G6PD-d | G6PD-d | Partially deficient | A- | EM/UM |
|  | 13 | G6PD-d | G6PD-d | Partially deficient | A- | IM |
|  | 14 | G6PD-d | G6PD-d | Totally deficient | WT | EM |
| 0.50 mg/kg | 15 | G6PD-d | G6PD-d | Partially deficient | A- | EM |
|  | 16 | G6PD-d | G6PD-d | Partially deficient | A- | EM |
|  | 17 | G6PD-d | G6PD-d | Partially deficient | A- | EM |
|  | 18 | G6PD-d | G6PD-d | Partially deficient | A- | IM |
|  | 19 | G6PD-d | G6PD-d | Partially deficient | A- | EM |
|  | 20 | G6PD-d | G6PD-d | Totally deficient | WT | IM |
|  | 21 | G6PD-d | G6PD-d | Partially deficient | A- | IM |
| Control | 22 | G6PD-n | G6PD-n | G6PD-n | WT | IM |
|  | 23 | G6PD-n | G6PD-n | G6PD-n | WT | IM |
|  | 24 | G6PD-n | G6PD-n | G6PD-n | WT | IM |
|  | 25 | G6PD-n | G6PD-n | G6PD-n | WT | EM |
|  | 26 | G6PD-n | G6PD-n | G6PD-n | WT | IM |
|  | 27 | G6PD-n | G6PD-n | G6PD-n | WT | IM |
|  | 28 | G6PD-n | G6PD-n | G6PD-n | WT | IM |

^1^ G6PD-n = G6PD-normal; G6PD-d = G6PD-deficient; WT = Wild-type at the G202A and A376G allele. G6PD testing results from semi-quantitative testing used for inclusion except for participant #10, who was also included although their blood sample for semi-quantitative testing was misplaced. Partially deficient (2.3 – 6.5 U/g Hb) and totally deficient (0 – 2.2 U/g Hb) were both considered as G6PD-d. G6PD-n was defined as > 6.5 U/g Hb.

^2^ PM = poor metabolizer, IM = intermediate metabolizer, EM = extensive metabolizer, UM = ultrarapid metabolizer

Among adults in phase 1, there were 12 *CYP2D6* intermediate metabolizers, 12 normal metabolizers, 1 normal / ultra-rapid metabolizer, and no poor metabolizers. One third (6/18) of G6PD-d participants were intermediate metabolizers, as compared to the G6PD-n group where nearly all (6/7) participants were intermediate metabolizers (Fisher’s exact *P*=0·07, Supplemental table S2), a borderline significant correlation. Exploratory ad-hoc analyses suggest that the distribution of *CYP2D6* genotypes did not differ significantly between the G6PD-deficient treatment groups (Fisher’s exact *P*=0·72). The within-person percent change in Hb following treatment with primaquine was not associated with CYP2D6 status (analysis not shown).

Table S2. *CYP 2D6* genotyping results by primaquine treatment group among adults only*

| Treatment group | 0.40 mg/kg | 0.45 mg/kg | 0.50 mg/kg | G6PD-normal 0.50 mg/kg | Total |
| --- | --- | --- | --- | --- | --- |
| Intermediate Metabolizer  N (%) | 1 (8) | 2 (17) | 3 (25) | 6 (50) | 12 |
| Normal Metabolizer  N (%) | 3 (25) | 4 (33) | 4 (33) | 1 (8) | 12 |
| Normal/Ultra-rapid Metabolizer  N (%) | 0 (0) | 1 (100) | 0 (0) | 0 (0) | 1 |

*For all individuals included in primary outcome analysis

In part 2, all participants with SNPs 202A on genotyping tested as partially G6PD-d (intermediate deficiency) using semi-quantitative assessment. Four participants (#29, #30, #38 and #39), were classified as partially G6PD-d according to semi-quantitative testing, and one was classified as totally deficient (#43), but did not have SNP 202A during genotyping.

Table S3. Part 2 Results of G6PD testing by individual participant

| Group / Participant ID# | | G6PD testing^1^ | | | |
| --- | --- | --- | --- | --- | --- |
|  |  | Qualitative (screening) | | Semi-quantitative | Genotyping (SNP 202A) |
|  |  | Carestart | OSMMR |  |  |
| 0.40 mg/kg G6PD-n 11-17 years of age | 31 | G6PD-n | G6PD-n | G6PD-n | WT |
|  | 32 | G6PD-n | G6PD-n | G6PD-n | WT |
|  | 35 | G6PD-n | G6PD-n | G6PD-n | WT |
|  | 36 | G6PD-n | G6PD-n | G6PD-n | WT |
|  | 39 | G6PD-n | G6PD-n | G6PD-n | WT |
|  | 40 | G6PD-n | G6PD-n | G6PD-n | WT |
|  | 42 | G6PD-n | G6PD-n | G6PD-n | WT |
| 0.40 mg/kg G6PD-d,  11-17 years old | 29 | G6PD-d | G6PD-d | Partially deficient | WT |
|  | 30 | G6PD-d | G6PD-d | Partially deficient | WT |
|  | 33 | G6PD-d | G6PD-d | Partially deficient | A- |
|  | 34 | G6PD-d | G6PD-d | Partially deficient | A- |
|  | 37 | G6PD-d | G6PD-d | Partially deficient | A- |
|  | 38 | G6PD-d | G6PD-d | Partially deficient | WT |
|  | 41 | G6PD-d | G6PD-d | Partially deficient | WT |
| 0.40 mg/kg G6PD-n,  5-10 years old | 47 | G6PD-n | G6PD-n | G6PD-n | WT |
|  | 48 | G6PD-n | G6PD-n | G6PD-n | WT |
|  | 49 | G6PD-n | G6PD-n | G6PD-n | WT |
|  | 51 | G6PD-n | G6PD-n | G6PD-n | WT |
|  | 52 | G6PD-n | G6PD-n | G6PD-n | WT |
|  | 54 | G6PD-n | G6PD-n | G6PD-n | WT |
|  | 56 | G6PD-n | G6PD-n | G6PD-n | WT |
| 0.40 mg/kg G6PDd,  5-10 years old | 43 | G6PD-d | G6PD-n | Totally deficient | WT |
|  | 44 | G6PD-d | G6PD-d | Partially deficient | A- |
|  | 45 | G6PD-d | G6PD-n | G6PD-n | WT |
|  | 46 | G6PD-d | G6PD-n | G6PD-n | WT |
|  | 50 | G6PD-d | G6PD-d | Partially deficient | A- |
|  | 53 | G6PD-d | G6PD-d | Partially deficient | A- |
|  | 55 | G6PD-d | G6PD-d | Partially deficient | A- |

^1^ G6PD-n = G6PD-normal; G6PD-d = G6PD-deficient; WT = Wild-type at the G202A allele.

1. Malaria infection detected by PCR at baseline, and by blood smear and symptoms on followup

At the screening visit, although no participants were symptomatic for malaria and all were smear negative, PCR analysis of stored blood samples from the screening visit revealed that 41% (21/51) of participants had asymptomatic malaria parasitemia. During follow-up, a total of 14 participants (27%) tested positive for malaria on blood smear at one or more visit days, with 4 of these 14 being symptomatic and treated for malaria during follow-up (two in the 0.40 mg/kg group, and two in the 0.45 mg/kg group).

Table S4. Malaria infection detected by PCR, blood smear, or clinical diagnosis at screening and follow-up

| Group / Participant ID# | | Screening | | | Followup | |
| --- | --- | --- | --- | --- | --- | --- |
|  |  | Asymptomatic malaria (PCR) | | | Blood smear parasite detection | Symptomatic malaria diagnosis (clinical assessment) |
|  |  | Pf | Pm | Po |  |  |
| 0.40 mg/kg G6PD-d adults | 4 | x |  |  | Day 28 |  |
|  | 5 |  |  |  | Day 14 |  |
|  | 6 | x |  |  | Day 1, 3, 4, 5, 6, 7 | Day 6 |
|  | 7 | x |  |  | Day 28 | Day 28 |
| 0.45 mg/kg G6PD-d adults | 9 | x |  |  |  |  |
|  | 10 |  |  |  | Day 28 | Day 28 |
|  | 11 | x | x |  | Day 9, 14 | Day 28 |
|  | 12 | x |  |  | Day 6, 8, 9, 10, 14 |  |
|  | 13 | x |  |  |  |  |
|  | 14 | x |  |  |  |  |
| 0.50 mg/kg G6PD-d adults | 16 | x |  |  | Day 6, 9 |  |
|  | 19 |  |  |  | Day 14 |  |
|  | 20 |  |  | x |  |  |
|  | 21 |  |  |  | Day 10 |  |
| 0.50 mg/kg G6PD-n adults | 22 | x |  |  | Day 28 |  |
|  | 23 | x | x |  |  |  |
| 0.40 mg/kg G6PD-d boys (age 11-17 years) | 30 | x |  |  | Day 1, 3, 14 |  |
|  | 41 | x |  |  | Day 14 |  |
| 0.40 mg/kg G6PD-d (age 5-10 years) | 43 | x |  |  | Day 5 |  |
|  | 44 | x |  |  |  |  |
|  | 53 | x |  |  |  |  |
|  | 55 | x |  |  |  |  |
| 0.40 mg/kg G6PD-n (age 5-10 years) | 48 | x |  |  |  |  |
|  | 49 | x |  |  |  |  |
|  | 51 | x |  |  |  |  |

G6PD-d = glucose-6-phosphate dehydrogenase (G6PD) deficient, G6PD-n = G6PD normal, Pf = *P. falciparum*, Pm = *P. malariae*, Po = *P. ovale*

1. Ad-hoc analyses investigating potential correlation between within-person changes in hemoglobin concentration and malaria infection. In ad-hoc linear regression analysis, we found no association between the within-person change in hemoglobin concentration during follow-up and malaria status, defined by PCR at screening, or blood smear during follow-up, or being symptomatic for malaria during any follow-up visit (analyses not shown).

Figure S3.

Supplemental Figure 2. Hemoglobin concentration (g/L) in G6PD-deficient men with and without malaria over 28 days of follow-up: absolute hemoglobin (S2a), and within-person percent change from baseline (S2b)

Boxplot key: median (line) IQR (box), and range (whisker).

* Within-person percent change in hemoglobin was significantly higher in malaria-infected, G6PD-d individuals as compared to non-infected individuals only on day 1 (p=0.035).

1. References

1. Cappellini MD, Fiorelli G. Glucose-6-phosphate dehydrogenase deficiency. *Lancet* 2008; **371**: 64-74.

2. Gaedigk A, Simon SD, Pearce RE, Bradford LD, Kennedy MJ, Leeder JS. The CYP2D6 activity score: translating genotype information into a qualitative measure of phenotype. *Clin Pharmacol Ther* 2008; **83**(2): 234-42.

3. St Jean PL, Xue Z, Carter N, et al. Tafenoquine treatment of Plasmodium vivax malaria: suggestive evidence that CYP2D6 reduced metabolism is not associated with relapse in the Phase 2b DETECTIVE trial. *Malar J* 2016; **15**: 97.

4. Johnson MK, Clark TD, Njama-Meya D, Rosenthal PJ, Parikh S. Impact of the method of G6PD deficiency assessment on genetic association studies of malaria susceptibility. *PLoS One* 2009; **4**(9): e7246.

5. Plowe CV, Djimde A, Bouare M, Doumbo O, Wellems TE. Pyrimethamine and proguanil resistance-conferring mutations in Plasmodium falciparum dihydrofolate reductase: polymerase chain reaction methods for surveillance in Africa. *Am J Trop Med Hyg* 1995; **52**(6): 565-8.

6. Schwartz A, Baidjoe A, Rosenthal PJ, Dorsey G, Bousema T, Greenhouse B. The Effect of Storage and Extraction Methods on Amplification of Plasmodium falciparum DNA from Dried Blood Spots. *Am J Trop Med Hyg* 2015; **92**(5): 922-5.

7. Hsiang MS, Lin M, Dokomajilar C, et al. PCR-based pooling of dried blood spots for detection of malaria parasites: optimization and application to a cohort of Ugandan children. *Journal of clinical microbiology* 2010; **48**(10): 3539-43.
